# Excision of a Portion of the Lower Jaw

**Published:** 1871-03

**Authors:** 


### ARTICLE VIII.

### *Excision of a Portion of the Lower Jaw.*

Dr. Forbes exhibited the patient and said:—

The case of the young woman now before the College is one of excision of one-half of the lower jaw-bone, with the exception of the condyle and coronoid process. The ablation was from the symphysis to the sigmoid notch on the left side.

The operation was performed just a year ago, and the woman is now in the enjoyment of excellent health. The sinking of the cheek is but little and the deformity is slight as the Fellows of the College will observe only a very small linear cicatrix from my excision, extending from the inferior margin of the symphysis along the under surface of the body to the posterior aspect of the angle of the bone.

H. B., aged 23 years, unmarried; works at a sewing machine; came in the Episcopal Hospital on the 9th of February, 1869.

Three years previous to her entrance she had observed a lump on the lower part of the gum of the left side of the lower jaw which she attributed to some decayed teeth, but having the teeth removed the lump did not disappear—on the contrary, it soon got larger. It gave her but little pain, and grew slowly, when in September, 1868, she observed some "growths" coming up from the jaw where the teeth were formerly lodged and which had been removed nearly a year previous. Early in November 1868, she went to the clinic of the University of Pennsylvania, and a portion of the tumor was removed through an incision extending along the inferior lateral surface of the bone. The wound healed, leaving an elevated iine or cicatrix. Soon after this she entered the Episcopal Hospital, and when I came on duty in

April the tumor was observed to be increasing. It had been three years in attaining its growth; it was comparatively painless; the tissues covering it remained healthy, though greatly distended; the absence of all glandular disease and of all cachexia, and any tendency to ulceration; its situation and the freedom from resentment of any injury, although a portion of it had been removed some months previous, all these points indicated its benign nature, and the propriety of recommending its removal. Accordingly at a consultation held the first week in May it was so decided. The patient was anxious for the operation, and it was at once performed.

Ether being administered the left inferior incisor was removed, and an incision was made with a bistoury under the chin just behind the symphysis and the knife carried up close behind the bone on its concave aspect until it appeared behind the gap made by the drawn tooth; a chain was then passed up through this opening by means of an eyed probe armed with a ligature having the saw attached. The jaw-bone was then bisected at the symphysis, the tongue being held out of the way by means of a thread passed through its tip. An incision was then made from the under surface of the symphysis along the inferior border of the bone to the posterior aspect of the angle; the facial artery was cut and secured at both ends, the soft parts with as much of the periosteum as possible were elevated; a similar incision was made nearly parallel to this so as to include the rough linear cicatrix of the former operation, and the parts detached as rapidly as possible. The inferior dental nerve and artery where they enter the posterior dental foramen were severed and the artery secured. The chain saw was now passed around the middle of the ramus of the bone and the end near the symphysis grasped with a strong pair of forceps, was held by an assistant, and the section of the bone removed.

The gap thus made was large—six ligatures in all were applied and the general oozing arrested by bathing the part with equal parts of alcohol, laudanum, and water. The amount of blood lost was believed to be about four ounces. The lips of the wound were approximated and held by leaden wire, and the cavity packed through the mouth with pledgets of lint. A dressing of cerate was applied and sustained by a bandage, the ligatures being brought out of the angle of the mouth. The patient reacted well, and an anodyne being administered she was removed to the ward. The anodyne was repeated frequently, and she was sustained by liquid nourishment. The sutures were removed the third day; the ligatures came away in due time, and in four weeks she left the house well.

On examining the tumor it was found inclosed by the body of the bone, having distended its walls, particularly the outer one, until they had become exceedingly thin and destroyed in two places, as may be observed in the specimen in the bottle. Its boundary was well defined, though it was not in a capsule ; its centre was soft and almost gelatinous, and of a grayish-white colour ; and every part of it was easily broken up. On examination under the microscope, cells, angular and attenuated like caudate cells, having dotted contents, nuclei and nucleoli, and free nucleoli, as observed by Mr. Paget in his description of myeloid tumours, were readily seen. With Mr. Paget, I prefer to believe, however, that the true nature of morbid growths generally is to be apprehended more correctly by examining them as living things, and modes of life rather than conditions of structure determine me in classifying them.

The tumour was evidently a myeloid one, the nature of which has been well described by Mr. Paget, and more recently by Mr. Cristopher Heath in his excellent work on Injuries and Diseases of the Jaw, the Jacksonian Prize Essay of the Royal College of Surgeons of England published in 1868.—*Transactions of College of Physicians.*

